# Evaluation of the Influence of Flavor Characteristics of Cooked Bacon with Different Sterilization Methods by GC-IMS Combined with HS-SPME-GC-MS and Electronic Nose

**DOI:** 10.3390/foods11223547

**Published:** 2022-11-08

**Authors:** Ruixiao Wu, Chunjie Yang, Linjie Xi, Tian Wang, Ju Zhang, Liping Kou, Wu Ding

**Affiliations:** College of Food Science and Engineering, Northwest A&F University, Xianyang 712100, China

**Keywords:** bacon, electron beam irradiation, ion mobility spectrometry, volatile compounds

## Abstract

This study investigated the impact of high pressure and temperature (HTHP) and electron-beam irradiations (3, 5, 7, and 9 kGy) using differences in two sterilization methods on the volatile compounds and sensory characteristics of cooked bacon. It showed that 7 and 9 kGy of irradiation caused a significant reduction in species of volatile compounds and sensory features, but the concentration of total ketones, alcohols, aldehydes, acids and aromatic hydrocarbons significantly increased at 9 kGy. Samples treated with a dose of less than 5 kGy did not change volatile compounds and sensory properties. High-temperature–high-pressure conditions could greatly impact the concentrations of volatile compound species and sensory traits. The electronic nose effectively detected the flavor difference in different sterilization methods. Fingerprinting showed that HTHP and 9-kGy-treated groups were significantly different from other treatments. This study inferred that 5 kGy might be optimal for maintaining the original flavor and sensory properties of cooked bacon.

## 1. Introduction

Bacon is a traditional Chinese delicacy. It refers to products typically made of pork meat that have undergone smoking and salting. This is not only a protective process involving the production of antioxidants and antibacterial ingredients, which is also an effective mechanism during flavor formation through the smoking process [1], but is also an effective mechanism during flavor formation through the smoking process. Apart from this, unique flavors are an essential reference for evaluating the quality of bacon [2].

In the processing of meat products, the existing sterilization technologies for cooked meat are divided into thermal sterilization and non-thermal sterilization. However, thermal sterilization affects the texture and flavor of meat products significantly. Irradiation treatment (X-ray, γ-ray and electron beam) is a safety measure in non-thermal sterilization technology that can extend the storage period of meat products [3]. Electron beam (EB) sterilization is a relatively short process and is safer than gamma rays because it is not radioactive and has a low effect on flavor [4].

To date, some studies were conducted on the application of EB technology to cooked meat products [5]. Many researchers investigated the effects of irradiation at different doses on the physicochemical properties and flavors of different meat products [6,7]. However, studies on the flavor changes of cooked bacon after using different sterilization methods were few.

In recent years, gas chromatography-mass spectrometry (GC-MS) and electronic nose (E-nose) have become mainstream approaches to analyze the volatile compounds of meat production [8]. GC-MS is applied to perform a qualitative analysis of volatile compounds and conduct quantitative analysis with high sensitivity. E-nose technology is a rapid detection technology that can quickly identify the categories of volatile substances in meat with sensors. However, these two technologies have certain disadvantages. The presence of isomers in mass spectrometry during separation could impact the separation effect [9]. E-nose technology can only classify volatile compounds approximately. Ion mobility spectrometry (IMS) is a gas-phase electrophoresis technique that can determine compounds present in trace quantities [10]. The detection principle of IMS to distinguish flavor compounds is based on the difference in gas-phase ion mobility in drift tubes under an atmospheric electric field [11]. This technique combines the separation ability of gas chromatography and the advantages of rapid and sensitive ion transfer spectroscopy [12]. It has been applied to detect flavor substances in meat products and other relevant field research in recent years [2,11]. However, few studies used the combined application with E-nose and GC-MS technology to investigate the changes in the flavor of bacon by different sterilization methods.

The primary purpose of this study was to explore the changes in volatile flavor compounds in cooked bacon by two sterilization methods at high temperature and pressure and under EB irradiation. E-nose and GC-MS combined with GC-IMS were selected to analyze the flavor compounds and determine the critical odor active compounds in cooked bacon. Therefore, the results obtained provide a better comprehensive and detailed understanding of the effects of different sterilization methods on the characteristic volatile flavor compounds.

## 2. Material and Methods

### 2.1. Bacon Preparation and Storage Conditions

The bacon sample was purchased from Zhenba County, Hanzhong City, Shaanxi province. The bacon was processed according to traditional procedures. The marinations were as follow: 1000 mL of baijiu (Chinese liquor) was uniformly spread on the surface of the fresh pork, and then immersed with 4% dry salt (g/100 g fresh sample) at 15–20 °C for 3 days. Afterwards, the samples were intermittently cold-smoked using Cyclobalanopsis glauca wood for 15 days.

The bacon was stewed in boiling water for 3 h and the final internal temperature reached was 72 °C, as measured with a thermometer. Then, the excess fat tissue was removed. Excess moisture from the surface was eliminated through the drying process, i.e., air-dried in a blast drying box, with the temperature controlled to 30 °C and the upper and lower layers exchanged every 6 h, for a total 12 h drying process. Then, all of the lean bacon was cut into 3 mm slices and divided into three groups. A total of 360 bacon slices (50 × 25 mm^2^) were obtained and individually packaged in vacuum bags (nylon/polyethylene vacuum bags). The control group (Control) of samples was not subjected to any treatment; one group (HTHP) was subjected to sterilization at a high temperature and pressure (121 °C, 0.1 MPa) for 15 min. The remaining four groups were sterilized using EB irradiation (3, 5, 7, and 9 kGy). The samples were processed and transferred to a refrigerator at 4 ± 1 °C in the laboratory.

### 2.2. Radiation Process

The samples were placed in a polystyrene foam box with ice bags to maintain a constant temperature (2–4 °C) before irradiation. The irradiated samples were sent to Yangling Nuclear Power Radiation Technology Co., Ltd. (Xianyang, China) for EB irradiation. The E-beam irradiation source was organized using a radio-frequency accelerator (energy 2.5 MeV and beam power 40 kW) to treat samples at irradiation doses of 3, 5, 7, and 9 kGy. The irradiation lasted for 2 h. Alanine dosimeters were placed at the top and bottom of each sample to determine the absorbed dose within ±5% of the targeted dose. All samples were sent to the laboratory immediately after irradiation and stored in a refrigerator at 4 °C.

### 2.3. Electronic Nose Measurement

The E-nose analysis was conducted using a PEN3 E-nose sensor equipped with 10 different detectors according to the method of [13]. A cooked bacon sample was cut accurately, weighed (1 g) and placed in a 20 mL headspace glass enrichment bottle; the carrier gas was clean air. The experimental protocol test samples were divided into 6 measurement groups with 10 parallel samples in each treatment group. The instrument manual was strictly followed during evaluating all samples using standard procedures. Each sample measurement was sealed with a rubber plug bottle cap after 270 s and enriched for 30 s to equilibrate the headspace volatiles of bacon. Subsequently, the air supply needle was inserted completely, and the collection needle was inserted partially when the time was 300 s; the collected time for the detection was set as 60 s. After the sampling process, the entire E-nose instrument was thoroughly cleaned with air for 300 s after each detection. Therefore, the following treatment sample was not affected by the previously sampled gas residues.

### 2.4. Analysis of Volatile Compounds

Volatile compounds in cooked bacon were extracted and analyzed according to with some modifications [2]. The extraction of volatile compounds from bacon samples manufactured using different sterilization methods was performed by headspace solid-phase microextraction (HS-SPME). The identification of volatile organic compounds was conducted by GC-MS. Then, 3.0 g of minced bacon was taken into 20-mL headspace sample vials. Further, 2.5 μL of 2-methyl-3-heptanone (3 μg/mL, dissolved in hexane) was added as the internal standard. The samples were extracted at 60 °C for 30 min, and the fiber-absorbed compound was decomposed at 260 °C for 15 min. Volatile components were separated using the RTx-5 capillary column. Helium was used as the carrier, and the linear velocity was 36 cm s^−1^. The temperature of the oven chromatograph was maintained at 40 °C for 5 min and was then increased from 40 °C to 240 °C within 15 min. The C6-C20 n-alkanes were determined under the same conditions to calculate the linear retention index. Three replicates (triplicate) of each treatment group were used for the analysis by comparing their mass spectra and retention times with those available in the local mass spectrum library to identify volatile compounds in the experimental groups. The relative contents of volatile compounds were calculated using the peak area for each detected compound divided by the peak area of the standard compound (expressed as μg/kg).

### 2.5. Odor Threshold

The odor activity value (OAV) represents the contribution of volatile compounds to the taste, calculated by dividing the concentration of volatile compounds by its threshold. The odor thresholds of these compounds were derived from the Compilation of Olfactory Thresholds of Compounds (second edition of the original book).

### 2.6. GC-IMS Analysis of Volatile Compounds

The GC-IMS Volatile compounds analysis in cooked bacon was conducted according to Xing et al. [14]. The GC-IMS analysis was performed using a GC in conjunction with the IMS instrument (Flavourspec^®^-G.A.S. Dortmund Company, Dortmund, Germany). MXT-5 implemented chromatographic separation using the L-15 m internal diameter column (15 m × 0.53 mm × 4 μm). Then, 2 g minced bacon was put into a 20 mL headspace sample bottle. Three replicate samples were collected for each treatment condition. The mean value was computed across three samples. Further, 3 μg/mL 2-methyl-3-heptanone (50 μL) was added to the samples as the internal standard and incubated at 80 °C for 20 min. The injection volume of the instrument was 200 μL, and the sample gas temperature was kept at 80 °C for 30 min. Nitrogen was transferred as the detection carrier gas to the capillary column of MXT-5 (15 m × 0.53 mm) whose column temperature was maintained at 60 °C. The flow velocity was changed every 10 min at 2.0, 10, 100 and 150 mL/min. The final result was an average of three replicates. The outcomes for GC-IMS data analysis were presented as fingerprint chromatograms of the individual treatment groups using a G.A.S. software suite called Laboratory Analytical Viewer.

### 2.7. Sensory Evaluation

The sensory evaluation of the cooked bacon products was conducted following the method described by Du et al. with some modifications [15]. The training of sensory study volunteers selected 15 motivated and energetic members from faculty, staff and graduate students (*n* = 15, aged 20–40, 8 male, 7 female). Six sessions were taken. These selected assessors presented and reviewed the scoring standards of the characteristic samples at a face-to-face meeting. Furthermore, they received about 2 h of training during each session. Redness, hardness, smokiness, flavor, off-odor and overall acceptability of bacon were assessed using a 7-point line scale: the “score = 1,” the “score = 4,” and the “score = 7” represented the degree of redness including light pink, moderate redness, and dark red, respectively. For hardness, 1 = tough and 7 = tender; for smokiness, the “score = 1” indicated non-smoky, the “score = 4” indicated average smoky, and the “score = 7” indicated intense smoky. For bacon flavor, 1 = nondetectable and 7 = intensely smoked bacon flavor; for off-odor, the “score = 1” indicated non-odorous, the “score = 4” indicated average odorous, and the “score = 7” indicated strong off-odor. For overall acceptability, the “score = 1” indicated unacceptable, and the “score = 7” indicated exclusively receptive. The samples were placed on a transparent dish codified with randomized 3-digit numbers. In each data acquisition session, all samples were measured in 1 day to guarantee optimal comparability among the samples. Each member was provided with drinking water to cleanse their mouths of any food residues before evaluating subsequent samples and requested seated rest breaks of 8–10 min for sensory recovery after the sensory assessment were allowed.

### 2.8. Data Analysis

Origin 2021 software package was used for data analysis. Analysis of variance was performed (*p* < 0.05) among the means using the Tukey procedure. The results were expressed as the mean values ± standard error (SE). The response values of each electronic sensor were analyzed by principal component analysis (PCA) to evaluate the potential of E-nose to discriminate among the flavor profiles of cooked bacon after different sterilizations. The volatile compound results were expressed as the mean values ± SE. The correlation analysis results were plotted in the form of a heatmap using the corrplot package in R software. The GC-IMS chromatograms were presented and analyzed by gallery plot analysis and GC × IMS Library Search supported by G.A.S. (FlavourSpec^®^ in the G.A.S. Department of Shandong HaiNeng Science Instrument Co., Ltd., Jinan, China).

## 3. Results and Discussion

### 3.1. E-Nose Analysis

**Figure 1:**
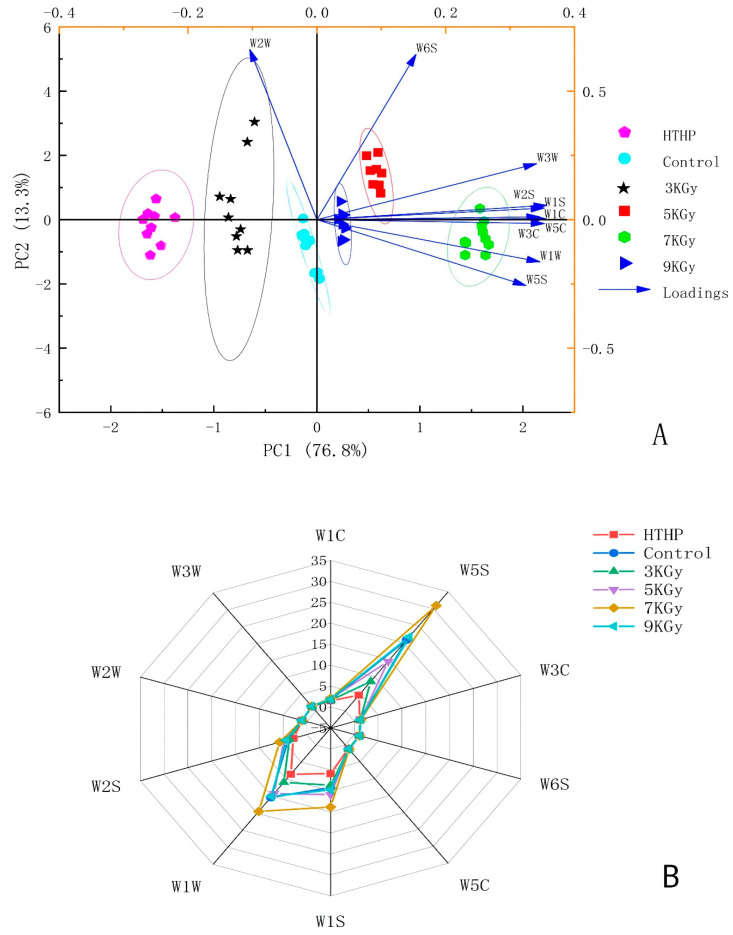
Principal component analysis (**A**) and radar chart (**B**) of electronic nose data for cooked bacon with different sterilization methods. Control: non- high pressure and high-temperature and non-irradiated bacon; HTHP: high pressure and temperature treated bacon; 3 kGy: 3 kGy-dose irradiated bacon; 5 kGy: 5 kGy-dose irradiated bacon; 7 kGy: 7 kGy-dose irradiated bacon; 9 kGy: 9 kGy-dose irradiated bacon. A 95% Confidence Ellipse was exhibited in Figure 1A.

According to the radar chart in Figure 1A, the response value of bacon volatile compounds of sensors W5S, W1S, W1W and W2S had more significance and was dissimilar compared with that of sensors W1C, W3W, W2W, W2S, W5C, W6S and W3C. It indicated that distinct sterilization techniques had different effects on the flavor components of cooked bacon. High-dose (7 amd 9 kGy) irradiation might produce more nitrogen oxides compared with other treatments, including methane, sulfur-containing compounds, alcohols, aldehydes, ketones and pyrazines. The content of flavor compounds might significantly decrease at high temperature and pressure. Nitrogen oxides, sulfides and pyrazines produced at low doses (3 and 5 kGy) might be reduced. It had a minimal effect on the changes in the contents of alcohols, aldehydes, ketones and methane. The results indicated that the E-nose system could effectively distinguish aroma regions in cooked bacon. The PCA is a statistical instrument that can describe the differences compared with various sample treatments.

The space distribution of bacon aroma was analyzed by PCA according to the spatial distribution characteristics of bacon aromatic compounds [13]. The findings are illustrated in Figure 1B. PC1 and PC2 accounted for 76.8% and 13.3% of the total variance, and the first two compositions accounted for 90.1% of the total variance, which implied that the two principal components could reflect all the information characteristics of cooked bacon and also demonstrated various features of volatile odor composition. The high temperature combined with the high pressure and irradiation through four doses had their aroma districts. The E-nose could separate various processed samples. The variation between each group of bacon samples was mainly found on PC1. The bacon sample groups exposed to 5, 7 and 9 kGy of radiation were mainly clustered in the positive axis of PC1, which correlated with the rest of the sensors except sensor W2W. Further, the samples subjected to 3 kGy radiation, samples exposed to high temperature and pressure and controls were clustered in the PC1 negative axis, which correlated with the sensor W2W. Therefore, the content of sulfur compounds was perhaps regarded as the marker to differentiate the sterilization methods of instant bacon. Consequently, the E-nose could be used as a tool to distinguish the effects of diverse sterilization treatments on the flavor characteristics of cooked bacon. However, this technology could not determine volatile compounds accurately.

### 3.2. Volatile Compounds and OAV Analyses

The relative concentrations of volatile flavor compounds detected in cooked bacon after different sterilization treatments are shown in Table 1. A total of 68 flavor compounds were identified and quantified in samples, comprising 13 alcohols, 11 ketones, 7 aldehydes, 5 esters, 8 phenols, 3 acids, 4 furans, 6 aromatic hydrocarbons, 2 ethers, 5 terpenes and 4 other flavor compounds. A total of 53 volatile compounds were tested in the high-temperature and high-pressure group, and 10 volatile substances were compared with 43 substances in the control group, suggesting that high temperature and high pressure could produce more flavor compounds. Our results were consistent with the findings of [16], whereby the main flavor compounds include isoamyl alcohol, cineole, linalool, 1-nonanal, guaiacol and cis-Anethol. In the irradiation group, the main compounds of each treatment group include ethanol, isobutyl alcohol, isoamyl alcohol, linalool, guaiacol, cineole, 3-hydroxy-2-butanone and cis-anethol. Finally, 41 of the most volatile substances were detected in the experimental groups after sterilization using 5 kGy irradiation, whereby aromatic compounds included linalool, guaiacol and cis-Anethol also had the highest content compared with other irradiation groups.

Alcohols can be formed through glucose metabolism, lipid oxidation, amino acid decarboxylation and a dehydrogenation mechanism [17]. The total alcohol content was significantly lower in the high-temperature and high-pressure samples than in the control group. GC-MS results in each irradiation treatment group were consistent with E-nose determination. Higher concentrations of ethanol, isobutanol, isoamyl alcohol, eucalyptol and linalool were detected in the control group. It indicated that thermal treatment might cause protein hydrolysis to produce more amino acids, peptides and small-molecule compounds and promote the Maillard reaction [18]. In addition, no significant difference in the total alcohol content was observed between the control and the 5 kGy-treated group. It displayed the increasing significance of the role of the ethanol content with the increasing irradiation dose. A positive correlation was found between alcohols and lipid oxidation in cooked turkey meat after irradiation by Feng et al. [5]. The irradiation process generated a large number of free radicals that accelerated lipid oxidation through free radical chain reactions [19]. It showed that the concentration of isobutanol and isoamyl alcohol decreased first at 3 kGy and then increased with the increase in radiation dose. The samples during high-dose irradiation exhibited a higher response value at the E-nose sensor W2S. Still, no significant change was found in the concentrations of some flavor compounds. The concentration of some volatile compounds (n-hexanol, 1-octene-3-ol, and α-terpineol) even decreased with the increase in dose. It indicated that not only did irradiation generate some new volatile flavor compounds, but the volatile compounds already existing in the bacon treatment samples were also degraded [20]. According to the OAV shown in Table 2, the nine alcohols with OAV ≥ 1 were considered as the critical volatile flavor compounds in the cooked bacon, including isobutanol, isoamyl alcohol, eucalyptus, hexanol, 1-octene-3-ol, linalool, 1-octanol, furfuryl alcohol and (S)-(–)-α-terpineol. It showed that the treatment sample had more crucial volatile substances through irradiation, which played an important role in the flavor. The finding of this study was consistent with the finding of Kong et al. [21]. Furfuryl alcohol is a furan derivative. Based on a solid OAV, the main flavor compound contributors to the bacon sample were 1-octen-3-ol and linalool, which agreed with the findings of Zhang et al. [17]. 1-Octen-3-ol might attribute to the fruity aromas of the samples [22]. Linalool presented a unique aromatic odor mainly derived from spices such as cloves, orange peel, ginger and so forth [23].

Thirteen kinds of ketones were detected in treatment samples in this study. Ketones contributed to the formation of unique aromas such as fruit, wood and mushroom flavors [24]. Ketones were produced by the degradation of amino acids, oxidation or degradation of unsaturated fatty acids, fermentation of carbohydrates by different microorganisms and oxidation of β-keto acids [25]. For example, 3-hydroxy-2-butanone was produced by microbial fermentation [26]. In this study, 3-hydroxy-2-butanone was found in each treatment group with the highest concentration. The 5 kGy-treated group showed no significant difference in the concentration of total ketones compared with the control group. The findings on OAV in Table 2 indicated that 3-hydroxy-2-butanone, 2-pentanone and 2-heptanone contributed significantly to the flavor. The total concentration of ketones increased with the increase in the irradiation dose up to 7 kGy when they reached their maximal concentration. This finding was consistent with the response value of the E-nose W2S sensor. The oxidative deterioration of free fatty acids is a crucial pathway for 2-ketone formation [27]. The concentration of 2-pentanone was the highest at 9 kGy; it had ethereal, buttery, spicy and blue cheese aromas [28], 2-Heptanone was detected at the highest concentration in the high-temperature and high-pressure group, which was significantly different from that in the other treatment groups (*p* < 0.05). The flavor compounds had spicy and blue cheese aromas [29].

Aldehydes are important flavor compounds in meat products with a low odor threshold. They significantly contribute to the overall flavor of meat products. Five aldehydes were found in the blank control group. Four aldehydes were found in the high-temperature and high-pressure treatment groups, but the total concentration was lower than that in the control group (*p* < 0.05). No significant differences were observed in the three kGy-treated group compared with the 7 kGy-treated group and the control group with 5 kGy that had undergone irradiation treatment (*p* > 0.05); the total concentration was the highest in the 9 kGy-treated group. Nonanal was detected in all treated samples. It was possibly generated from oxidation [30], and had a greasy and sweet orange flavor [31]. The content of isovaleraldehyde in the irradiation treatment group increased with the increase in the irradiation dose. Hexaldehyde was detectable only in the control treatment group; it could be regarded as the indicator of the degree of secondary oxidation during lipid auto-oxidation [2]. The flavor compounds had a rancid odor at high concentrations, whereas fruity aroma and broth odor appeared at low concentrations [25]. Isovaleraldehyde and 2-methylbutanal were not detected in the control group. The present study demonstrated that high-dose irradiation (7 and 9 kGy) promoted the generation of these two aldehydes. The OAV values of three aldehydes, including isovaleraldehyde, octanal and nonanal, were greater than 1, indicating that these volatile flavor compounds contributed a lot to the flavor of the cooked bacon. The OAV isovaleraldehyde under 9-kGy irradiation in all samples was much higher than under other treatments. The lower OAV of nonanal under high-dose (7 and 9 kGy) irradiation indicated its contribution to the decrease in the aroma.

The primary formation mechanism of esters included those derived from esterification and combined acids with alcohols. Some microorganisms also promoted the generation of esters, leading to the production of volatiles that exhibited a floral and fruity odor [32], whereas their olfactory threshold was shallow. Thus, esters played an essential role in the overall aroma and flavor [26]. Five esters were identified in the present study. High-temperature and high-pressure treatment generated four esters with minor concentrations. Zou et al. showed that the hydrolysis of the ester led to the formation of acids and alcohols at a high temperature and high pressure [33]. This was the reason why more alcohols and acids were formed in the high-temperature and high-pressure group compared with the other treatment groups. 3,7-Dimethylocta-1,6-dien-3-yl 2-aminobenzoate was detected in each group in the present study. The treatment with two sterilization processes reduced the concentrations of the flavor compounds, which was significantly different from those in the blank control group (*p* < 0.05). The research result of this study was consistent with the findings of [21].

Phenolic compounds are the critical substances in the enrichment of meat smoke flavor. They comprise the intermediate products generated through lignin decomposition during the smoking process and the formation of guaiacol homologue through further reactions. Smoking can also positively promote phenol formation [34]. Eight phenolic species were found in the present study. Seven phenols were identified in the control group. GC-MS analysis detected a significantly decreased total concentration (*p* < 0.05) and quantified six phenolic compounds in the high-temperature and high-pressure group. Further, 4-methylphenol was detected only in the control group. Phenol and guaiacol were detected in all treatment groups, consistent with the results of Deng et al. [2]. The statistical analysis illustrated that high-dose irradiation (7 and 9 kGy) treatment significantly reduced the content of these two aromatic compounds compared with the other methods. The OAV value of two flavor compounds greater than 1 contributed significantly to the bacon aroma. Guaiacol was the main smoking component in smoked bacon, which highlighted the significant effect on flavor. Moreover, o-cresol and 4-ethyl guaiacol also contributed significantly to the flavor. No significant difference (*p* > 0.05) was found in the contents of these two compounds under non-high-dose (3 and 5 kGy) treatment conditions. The total phenolic compound concentration at 5 kGy was not significantly different from that in the control group.

A total of three acids were detected in this study, including acetate as the main component. The acetic acid content in the irradiated group increased with the irradiated dose compared with that in the non-irradiated controls. It revealed a significant difference (*p* < 0.05) in the content until the 9-kGy radiation was used. However, acetate had a higher odor threshold, which contributed less to the bacon flavor characteristics [35]. Benzoic acid and 2-acetyl-2-phenylhydrazine were detected only in high-temperature and high-pressure treatment samples. No significant difference (*p* > 0.05) in the isovaleric acid content was detected between the control and 5 kGy-treated groups. In addition, the volatile substances detected by GC-MS included furans and ethers. Anisole might be produced by the spices added in the process while cooking bacon. It inferred that 4-allyl-anisole contributed a lot to the flavor of the fresh fruits. Furan is a heterocyclic compound generated by the degradation of carbohydrates, thermal oxidation of lipids, and thermal degradation of thiamine in the Maillard reaction [36]. Hence, the content of furan increased significantly after high-temperature and high-pressure treatment. Furthermore, 2-pentylfuran had the highest content among the furan compounds. It exhibited a grassy and spicy aroma with a shallow threshold in water, indicating that it could contribute substantially to the flavor [16].

Aromatic compounds are essential components of flavor compounds in bacon. None of these volatile compounds were detected in the control group due to the high OAV value of toluene, which contributed the most to the bacon aroma. Toluene was formed probably by the auto-oxidation and oxidation of free tyrosine and showed a high correlation with hexanal [37]. The results showed that maximum aromatic compounds were detected in the high-temperature and high-pressure groups. They were formed during the thermal decomposition of hydrocarbons, fats and proteins [38]. Irradiated aromatic compounds in the treatment groups showed an increasing trend, which might be because the free radicals generated during the irradiation process promoted the substitution reaction of the benzene ring. The cooked bacon contained other volatile compounds. However, their classification was complicated. Water molecules were decomposed by irradiation to generate free water electrons, which would react violently with aromatic compounds and combine with oxygen to form hydrogen peroxide [19]; at the same time, free radicals also accelerated the formation of benzene and its derivatives. The amount of benzene and benzene derivatives in irradiated could be related to different amino acid profiles of meat [5]. Ahn et al. reported that phenylalanine produced more aromatic volatiles than tryptophan and tyrosine after irradiation (5 kGy) in aqueous model systems [39]. Pyrazine was a typical nitrogen-containing heterocyclic compound produced by the Maillard reaction [40]. 2,3,5,6-Tetramethylpyrazine presented a high OAV value that essentially contributed to the aroma. This volatile compound might be helpful in meat production to generate unique roasted, nutty and meat-, soil- and popcorn-like flavors during the heating process [41]. Terpene compounds in bacon usually come from the immersing step during processing, and hydrocarbons were the major radiolytic products in fat [21]. A total of five terpenes were identified in this study. The contents of terpenes were positively correlated with the addition of spices, such as black pepper, white pepper and garlic [42]. α-Pinene, g-Terpinene and Myrcene were also detected. The contents of α-Pinene in the Control, HTHP and 3 kGy treatment groups were significantly lower than those in the other irradiation treatment groups (*p* < 0.05). Among the irradiated bacon, the content of (4R)-1-methyl-4-(prop-1-en-2-yl) cyclohex-1-ene in the 5 kGy-treated group (18.25 μg/kg) was higher than those in other irradiation treatment groups (*p* < 0.05).

### 3.3. Correlation between E-Nose and GC-MS

**Figure 2:**
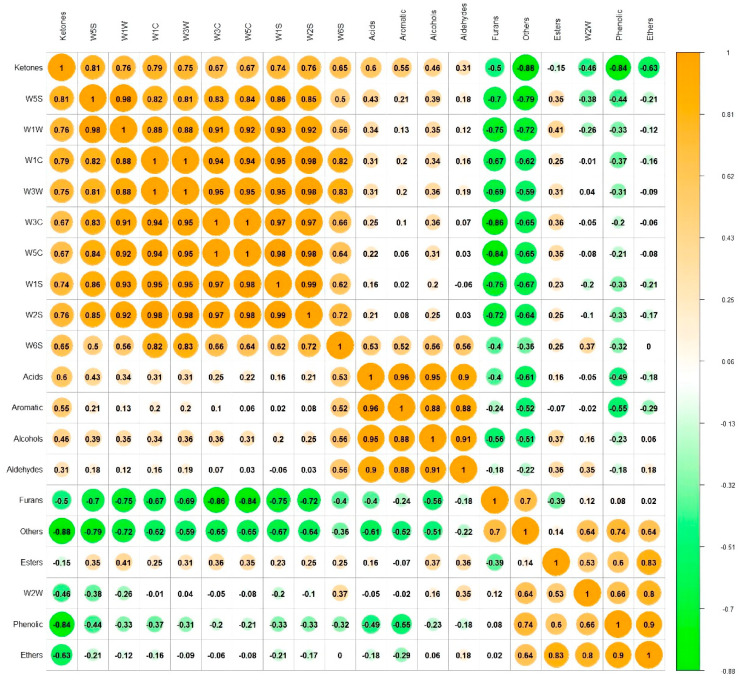
Correlations between the electronic nose and volatile flavor components.

The study next explored the larvaecious correlation between E-nose experiment responses and the concentration of volatile flavor compounds identified through GC/MS. The correlations between the response of E-nose sensors and the total concentrations of volatile compounds are presented in Figure 2. Furthermore, the *r* values represented the correlation coefficient corresponding to the degrees for evaluating the Pearson coefficient. The clustering analysis results are exhibited as a heatmap in Figure 2. The sensor W1C correlated negatively with the concentrations of furan and other flavor compounds detected by GC-MS (*r* = −0.67 and −0.62, *p* < 0.05). A positive correlation was found for ketones (*r* = 0.79, *p* < 0.05). The relative levels of W3C, W5C and W3W with furan compounds displayed a visible negative correlation (*r* = −0.86, −0.84, and −0.69, *p* < 0.05). The correlation of these observations of sensors W3C, W5C and W3W with the concentrations of ketones and other flavor compounds consistent with those of W1C (*p* < 0.05), indicating that the sensors W3C, W5C, W3W and W1C were sensitive to the aforementioned flavor substances. However, the concentrations of ketone species showed a negative correlation with sensors W2W (*r* = −0.46, *p* > 0.05) and positively correlated with other E-nose inductions. Du et al. found that the W1C, W3C, W5C, W1S and W2S sensors positively correlated with the concentrations of ketones [15]. This study revealed the positive correlation between the total concentration of esters and alcohols in cooked bacon and the response values detected by the E-nose. The association with esters was strong for W1W and W2W. The concentrations of phenolic compounds had a negative correlation with all sensors except W2W. However, the correlation of the concentrations of aldehydes with the E-nose was not significant. Yin et al. also reported that the correlation analysis revealed many aldehydes, ketones and phenols around the W1S, W2S, W6S, W1W and W2W sensors [13]. All of these experimental results demonstrated that the E-nose could discriminate between bacon types according to the response of volatile meat compounds. Similar correlation studies between the E-nose and GC-MS have been conducted in recent years [15]. However, this correlation only reveals the association between traits, not how one trait influences the other or is causative of the changes in the other.

### 3.4. Volatile Aroma Compound Profiling with GC-IMS

**Figure 3:**
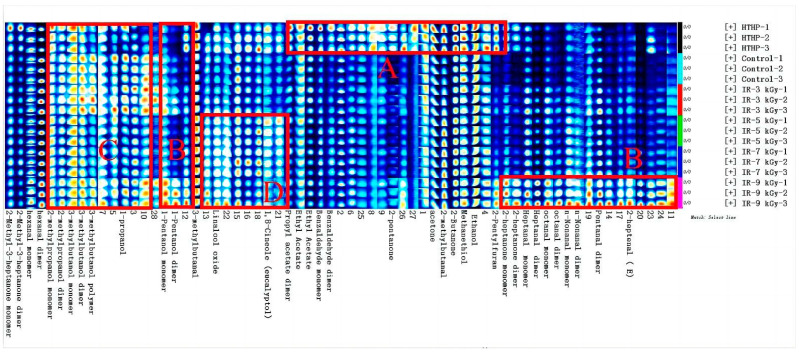
The fingerprints of cooked bacon with different sterilization methods are represented in the gallery plot by GC-IMS. Area A: High-pressure and temperature-treated bacon (HTHP); Area B: 9 kGy-dose irradiated bacon; Area C: Control: non-high pressure and high-temperature and non-irradiated bacon, 3 kGy-dose irradiated bacon; Area D: 5 kGy-dose irradiated bacon and 7 kGy-dose irradiated bacon.

The comparison was illustrated evidently by presenting all the identified substances according to the fingerprints in each treatment group of cooked bacon. Volatile flavor components in the HTHP group and other samples showed significant variations due to the highest concentrations of aroma compounds, as demonstrated in region A (Figure 3). The concentrations of propyl acetate, ethyl acetate, benzaldehyde, 2-pentanone, acetone, 2-methylbutanal, 2-butanone, methanol, ethanol and so forth were higher compared with that in other samples of main volatile compounds. Propyl acetate, benzaldehyde, methanethiol and 2-butanone were new substances not detected in the GC-MS analysis. Benzaldehyde was generated under the present Maillard reaction conditions. It was also formed by the oxidation or photochemical degradation of toluene or other hydrocarbons [16]. The formation of methylthiol depended on the degradation of sulfur-containing amino acids such as methionine, cysteine and cystine. With further reaction, it was converted into sulfhydryl groups, sulfoxides and disulfides. Meanwhile, typical flavor substances had “cabbage-like” or “burning feather” odors [43,44]. In addition, 2-butanone in irradiated samples was produced by ketone-converting secondary reactions after irradiation. Furthermore, 2-pentanone, 2-butanone and 2-heptanone presented ethereal, butter or spicy flavors [6,26].

In addition, the concentrations of volatile compounds irradiated with 3–9 kGy did not exhibit apparent regularity with the increase in the irradiation dose according to the fingerprint spectrum in Figure 3. The 9 kGy irradiation treatment group showed significant differences in the concentrations of volatile compounds compared with the other treatment groups. The substances in the region B of Figure 3 were especially much larger than those in other samples, such as 2-heptanone, heptanal, octanal, nonanal, valeraldehyde, 2-pentylfuran, 1-pentanol and so forth. The increase in the valeraldehyde content indicated that the high dose of EB promoted the exacerbation of lipid oxidation levels. This phenomenon was consistent with the research results of Feng et al. [6]. Heptanal is a high-level linear aldehyde generated during active lipid oxidation. It is a flavor compound in meat products with a low threshold and plays a vital role in meat flavoring [26]. The volatile compounds in the control and 3 kGy-treated groups were similar according to the fingerprint in region C of Figure 3. The contents of alcohols not detected in GC-MS, including propanol, 3-methyl-butanol and 2-methyl-propanol, were higher than those in the other treatment groups, indicating the influence of low-dose irradiation conditions on alcohols with no discernible changes. The ultimate flavor identification results of 5 kGy and 7 kGy treatment groups were relatively similar. The contents of 1,8-cineole and linalool oxide were the highest at 5 kGy and 7 kGy, respectively, in region D of Figure 3. Therefore, different sterilization methods inferred different degrees of protein and lipid oxidation reactions in cooked bacon. More new esters, ketones and alcohols were formed under high temperature and high pressure. The irradiated treatment samples had aldehydes and furan as flavor compounds.

### 3.5. Sensory Evaluation

**Figure 4:**
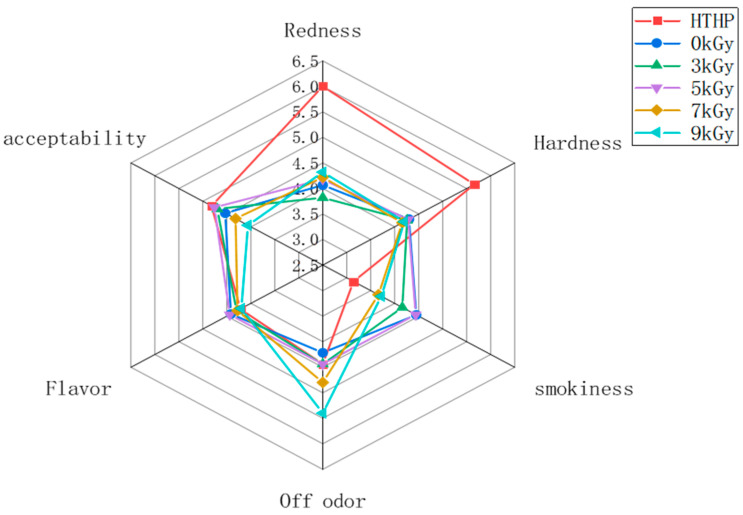
Radar chart of sensory evaluation data for cooked bacon with different sterilization methods. Control: non- high pressure and high-temperature and non-irradiated bacon; HTHP: high pressure and temperature treated bacon; 3 kGy: 3 kGy-dose irradiated bacon; 5 kGy: 5 kGy-dose irradiated bacon; 7 kGy: 7 kGy-dose irradiated bacon; 9 kGy: 9 kGy-dose irradiated bacon.

The analysis results of the sensory evaluation are shown in Figure 4. The redness value in the high-temperature and high-pressure treatment group was significantly higher than that in other treatment groups (*p* < 0.05). The differences between the irradiated group (3 and 9 kGy) and blank control groups were highly significant (*p* < 0.05). The 5-kGy and 7-kGy irradiated groups exhibited no significant difference compared with the control group (*p* > 0.05). The 3-kGy irradiated samples also had the lowest redness (*p* < 0.05). The increase in redness might be due to the effect of temperature during sterilization. The redness in the 3 kGy-treated group was significantly different from that in the other irradiated groups (*p* < 0.05), indicating that the degree of influence on bacon might be due to low-dose irradiation. Furthermore, the hardness values of bacon decreased significantly under high temperature and pressure, with no significant differences compared with the other groups. It suggested that high temperature and pressure might damage muscle fibers in meat products. Phenol and its derivatives are vital contributors to smoky odors in bacon products. The smokiness score of HTHP and high-dose irradiation (7 and 9 kGy) treatment samples were significantly reduced under these conditions (*p* < 0.05), which was consistent with the result of the total phenolic content through GC-MS analysis. The observed loss might be associated with phenolic compounds decomposed and degraded by high temperatures and irradiation (7 and 9 kGy). Additionally, an increase in apparent off-flavors was observed for the high-dose irradiated samples. It might be because high-dose irradiation promoted the decomposition of sulfur-containing amino acids [19]. The lowest overall acceptability was achieved at 9 kGy compared with the other groups.

## 4. Conclusions

In this study, we investigated the flavor compounds and sensory evaluation changes in cooked bacon after e-beam irradiation and high-pressure and high-temperature sterilization. High-temperature–high-pressure conditions greatly enhanced the production of volatile compounds, but the concentration of alcohols, aldehydes, acids and esters showed a downward trend (*p* < 0.05). The concentrations of major volatile compounds illustrated no significant difference for aldehydes, ketones, alcohols, phenols and acids between the control and the 5 kGy-treated group. Further, 7 kGy and 9 kGy irradiation caused a significant reduction in the number of species of volatile compounds. The results of the PCA indicated that the E-nose could discriminate between bacon samples through different sterilization methods. Moreover, the correlation analysis showed that the E-nose combined with GC-MS could distinguish between different bacon samples. GC-IMS technology could identify some aromatic substances not detected in GC/MS, and the distinction between aromatic substances was the most obvious at HTHP and 9 kGy. The sensory analysis demonstrated that high pressure and temperature increased redness and decreased the smoked bacon’s smokiness odor and hardness. Furthermore, 7 and 9 kGy exacerbated the levels of off-flavor. Moreover, 5 kGy of e-beam irradiation better guaranteed the product flavor. We therefore considered that varying the sterilization technology might have an impact on flavor compounds. Different flavor identification techniques could distinguish between bacon samples sterilized using different methods.

## Figures and Tables

**Table 1:** Contents (μg/kg) of volatile compounds in cooked bacon with different types of sterilization methods.

Volatile Compounds	R.T. (min)	LRI	Content (μg/kg^−1^) (Mean ± SE ^R^)					
			HTHP	Control	3 kGy	5 kGy	7 kGy	9 kGy
Alcohols								
Ethanol	8.69	813	5.09 ± 0.70 ^a^	12.48 ± 1.76 ^b^	13.22 ± 0.14 ^b^	13.12 ± 0.46 ^b^	18.22 ± 1.72 ^c^	41.25 ± 1.33 ^d^
Isobutyl alcohol	13.5	1096	0.38 ± 0.03 ^a^	1.44 ± 0.01 ^ab^	1.11 ± 0.34 ^ab^	1.81 ± 0.07 ^ac^	4.29 ± 0.59 ^b^	9.57 ± 1.35 ^c^
2-Cyclopentyl Cyclopentanol	16.69	1117	ND	4.14 ± 0.00 ^a^	ND	ND	ND	ND
Isoamyl alcohol	16.89	1131	10.35 ± 1.03 ^a^	33.52 ± 0.23 ^bc^	30.24 ± 0.41 ^bc^	43.60 ± 2.15 ^d^	56.04 ± 4.19 ^e^	84.85 ± 2.60 ^f^
Cineole	17.05	1144	21.67 ± 2.42 ^a^	34.54 ± 4.26 ^bc^	25.09 ± 1.69 ^bc^	40.60 ± 2.15 ^bd^	23.33 ± 2.99 ^a^	81.34 ± 3.54 ^e^
2-Undecanethiol,2-methyl-	20.20	1228	0.59 ± 0.00 ^a^	ND	ND	ND	ND	ND
Hexyl alcohol	21.48	1292	1.74 ± 0.24 ^a^	2.56 ± 0.06 ^bc^	2.40 ± 0.28 ^bc^	3.35 ± 0.03 ^d^	1.52 ± 0.18 ^a^	1.68 ± 0.00 ^a^
1-Octen-3-ol	24.34	1356	1.96 ± 0.29 ^a^	2.35 ± 0.22 ^a^	1.65 ± 0.21 ^ab^	2.41 ± 0.30 ^a^	0.50 ± 0.00 ^b^	ND
Linalool	27.10	1454	23.36 ± 2.62 ^a^	41.27 ± 0.50 ^b^	16.46 ± 4.35 ^a^	32.72 ± 1.53 ^bc^	3.13 ± 0.38 ^d^	6.95 ± 0.07 ^de^
1-Octanol”	27.37	1487	0.97 ± 0.09 ^a^	ND	0.66 ± 0.00 ^b^	1.44 ± 0.00 ^c^	ND	ND
Terpinen-4-ol	28.89	1620	5.99 ± 0.26 ^a^	9.01 ± 0.71 ^ab^	4.66 ± 0.34 ^a^	6.55 ± 0.65 ^ad^	ND	18.58 ± 0.00 ^c^
Furfuryl alcohol	30.33	1658	4.25 ± 0.15 ^a^	5.00 ± 0.38 ^a^	6.97 ± 1.02 ^a^	5.20 ± 0.03 ^a^	3.33 ± 0.41 ^a^	4.26 ± 0.60 ^a^
(S)-(-)-α-terpineol	31.19	1716	2.97 ± 0.39 ^a^	5.46 ± 0.32 ^b^	1.52 ± 0.42 ^a^	3.43 ± 0.37 ^a^	ND	ND
Total			79.36 ± 8.21 ^d^	151.75 ±8.45 ^b^	110.60 ± 10.47 ^c^	154.22 ± 7.75 ^b^	110.35 ± 10.47 ^c^	248.47 ± 9.49 ^a^
Aldehydes								
2-Methylbutyraldehyde	8.14	659	ND	ND	ND	ND	1.12 ± 0.00 ^a^	5.60 ± 0.60 ^b^
Isovaleraldehyde	8.24	672	ND	ND	ND	1.26 ± 0.00 ^a^	3.34 ± 0.00 ^a^	25.54 ± 0.91 ^b^
Hexanal	12.91	1085	ND	5.28 ± 0.00 ^a^	ND	ND	ND	ND
Heptaldehyde	16.33	1196	1.77 ± 0.00 ^a^	1.51 ± 0.00 ^a^	ND	2.50 ± 0.33 ^ab^	0.87 ± 0.10 ^a^	ND
Octanal	19.68	1202	2.73 ± 0.10 ^a^	1.99 ± 0.08 ^a^	2.77 ± 0.34 ^a^	3.92 ± 0.04 ^ac^	1.28 ± 0.00 ^b^	ND
1-Nonanal	22.92	1307	7.72 ± 1.00 ^a^	7.89 ± 0.46 ^a^	4.13 ± 0.02 ^b^	9.82 ± 0.41 ^a^	2.07 ± 0.00 ^bc^	2.14 ± 0.38 ^bd^
Decanal	25.98	1414	1.51 ± 0.10 ^a^	1.00 ± 0.10 ^b^	ND	2.45 ± 0.03 ^c^	ND	ND
Total			13.73 ± 1.21 ^c^	17.67 ± 0.64 ^b^	6.90 ± 0.37 ^d^	19.90 ± 0.81 ^b^	8.68 ± 0.44 ^d^	33.27 ± 1.88 ^a^
Acids								
Acetic acid	24.59	1365	2.84 ± 0.02 ^a^	4.00 ± 0.07 ^a^	3.78 ± 0.47 ^a^	3.69 ± 0.14 ^a^	5.63 ± 0.46 ^a^	14.62 ± 1.33 ^b^
Isovaleric acid	30.40	1678	ND	2.40 ± 0.10 ^a^	ND	2.02 ± 0.10 ^a^	ND	ND
Benzoic acid, 2-acetyl-2-phenylhydrazide	30.60	1686	1.11 ± 0.00 ^a^	ND	ND	ND	ND	ND
Total			3.95 ± 0.02 ^cd^	6.39 ± 0.18 ^b^	3.78 ± 0.47 ^d^	5.71 ± 0.24 ^b^	5.63 ± 0.46 ^bc^	14.62 ± 1.33 ^a^
Esters								
Ethyl acetate	7.51	789	ND	ND	ND	ND	6.68 ± 0.96 ^a^	8.16 ± 1.15 ^a^
Octadecanoic Acid, ethenyl ester	25.82	1408	0.67 ± 0.00 ^a^	ND	ND	ND	ND	ND
3,7-Dimethyl-1,6-octadien-3-yl 2-aminobenzoate	27.46	1468	2.31 ± 0.00 ^a^	22.31 ± 0.00 ^b^	3.58 ± 0.10 ^a^	17.53 ± 0.95 ^c^	1.05 ± 0.13 ^ad^	1.67 ± 0.21 ^a^
Ethyl caprate	29.60	1648	0.38 ± 0.10 ^a^	ND	ND	ND	ND	ND
Nerol acetate	31.66	1728	0.44 ± 0.10 ^a^	0.89 ± 0.10 ^a^	ND	ND	ND	ND
Total			3.78 ± 0.20 ^d^	23.20 ± 0.10 ^a^	3.58 ± 0.10 ^d^	17.53 ± 0.95 ^b^	7.73 ± 1.09 ^c^	9.83 ± 1.36 ^c^
Phenols								
Guaiacol	35.30	1724	9.01 ± 1.14 ^a^	13.52 ± 1.93 ^a^	11.62 ± 1.69 ^a^	14.73 ± 0.35 ^b^	3.09 ± 0.28 ^c^	2.93 ± 0.39 ^d^
2-Methoxy-6-methylphenol	35.63	1760	0.61 ± 0.00 ^a^	6.92 ± 0.00 ^a^	ND	1.16 ± 0.14 ^a^	ND	ND
O-Cresol	37.95	1905	4.13 ± 0.17 ^a^	5.40 ± 0.81 ^a^	3.97 ± 0.27 ^a^	6.17 ± 0.18 ^a^	0.90 ± 0.11 ^b^	ND
Phenol	38.38	2027	7.00 ± 0.35 ^a^	9.20 ± 0.36 ^a^	9.98 ± 1.17 ^a^	10.66 ± 0.03 ^a^	2.49 ± 0.06 ^b^	4.21 ± 0.00 ^b^
4-Ethyl-2-methoxyphenol	38.42	2029	3.39 ± 0.10 ^a^	4.95 ± 0.00 ^a^	2.96 ± 0.10 ^a^	4.99 ± 0.00 ^a^	0.49 ± 0.00 ^b^	ND
2,3-Dimethylphenol	39.78	2098	0.68 ± 0.00 ^a^	ND	ND	ND	ND	ND
p-Cresol	39.79	2099	ND	1.19 ± 0.00 ^a^	ND	ND	ND	ND
Total			21.43 ±1.76 ^c^	41.17 ±3.09 ^a^	28.52 ±3.23 ^b^	41.78 ±0.70 ^a^	6.97 ± 0.45 ^d^	7.14 ± 0.39 ^d^
Furans								
2-Methylfuran	7.20	770	2.10 ± 0.00 ^a^	ND	ND	ND	ND	ND
2-Ethylfuran	9.08	836	1.58 ± 0.00 ^a^	ND	ND	ND	ND	ND
2-Pentylfuran	17.72	1193	14.77 ± 0.00 ^a^	ND	ND	0.86 ± 0.00 ^b^	ND	ND
2-Acetylfuran	26.48	1432	1.00 ± 0.14 ^a^	1.73 ± 0.00 ^a^	1.73 ± 0.24 ^a^	1.474 ± 0.00 ^a^	ND	ND
Total			19.45 ± 0.14 ^a^	1.73 ± 0.00 ^c^	1.73 ± 0.24 ^c^	2.34 ± 0.00 ^b^	ND	ND
Ketones								
Acetone	6.38	720	ND	ND	ND	ND	ND	2.16 ± 0.10 ^a^
2-Pentanone	9.84	881	ND	ND	1.61 ± 0.00 ^a^	1.68 ± 0.00 ^b^	1.62 ± 0.02 ^ab^	2.09 ± 0.03 ^c^
3-Eicosanone	14.83	1150	1.01 ± 0.10 ^a^	1.20 ± 0.10 ^b^	0.94 ± 0.10 ^c^	1.33 ± 0.10 ^d^	ND	ND
2-Heptanone	16.22	1193	2.16 ± 0.15 ^a^	0.73 ± 0.10 ^b^	0.66 ± 0.10 ^b^	0.98 ± 0.10 ^b^	0.52 ± 0.10 ^bc^	ND
2-Octanone	19.57	1196	0.64 ± 0.00 ^a^	ND	ND	ND	ND	ND
3-Hydroxy-2-butanone	19.81	1208	5.28 ± 0.38 ^a^	8.35 ± 1.05 ^a^	10.62 ± 1.01 ^a^	13.01 ± 0.02 ^ab^	45.00 ± 6.35 ^b^	34.523 ± 4.208 ^b^
Hydroxyacetone	20.40	1238	1.13 ± 0.14 ^a^	ND	ND	ND	2.08 ± 0.09 ^a^	4.08 ± 0.50 ^a^
Methylheptenone	21.23	1280	2.90 ± 0.27 ^a^	2.48 ± 0.35 ^a^	1.14 ± 0.15 ^a^	2.43 ± 0.30 ^a^	0.66 ± 0.00 ^a^	1.06 ± 0.15 ^a^
2-Cyclopenten-1-one,2-methyl-	22.55	1385	1.11 ± 0.20 ^a^	ND	ND	ND	ND	ND
2-Cyclopenten-1-one,2,3-dimethyl-	27.65	1474	1.66 ± 0.16 ^a^	2.46 ± 0.07 ^a^	2.48 ± 0.49 ^a^	2.74 ± 0.02 ^a^	0.53 ± 0.21 ^a^	ND
Acetophenone	30.61	1686	ND	1.70 ± 0.00 ^a^	ND	0.98 ± 0.00 ^a^	ND	ND
Total			15.89 ±1.41 ^b^	16.92 ± 2.78 ^b^	17.45 ±4.95 ^b^	23.13 ± 0.55 ^b^	48.33 ± 6.76 ^a^	43.91 ± 4.99 ^a^
Aromatic hydrocarbons								
Toluene	11.62	1021	ND	ND	1.34 ± 0.20 ^a^	2.82 ± 0.02 ^a^	2.58 ± 0.37 ^a^	4.00 ± 0.47 ^a^
1-Methyl-4-isopropylbenzene	19.09	1170	ND	ND	ND	4.78 ± 0.00 ^a^	ND	ND
m-isopropyltoluene	19.16	1174	2.58 ± 0.10 ^a^	ND	ND	ND	ND	48.29 ± 0.00 ^a^
Benzene,1,3-bis(1,1-dimethylethyl)	23.82	1338.	ND	ND	ND	0.49 ± 0.00 ^a^	ND	ND
3,4-Dimethoxytoluene	34.06	1626	0.75 ± 0.00 ^a^	ND	ND	ND	ND	ND
3-Hydroxy-4-methoxytoluene	35.77	1670	0.90 ± 0.10 ^a^	ND	ND	ND	ND	ND
Total			4.23 ± 0.20 ^a^	ND	1.34 ± 0.20 ^b^	8.09 ± 0.02 ^c^	2.58 ± 0.37 ^d^	52.29 ± 0.47 ^e^
Ethers								
4-Allylanisole	30.75	1692	0.99 ± 0.02 ^a^	2.81 ± 0.41 ^a^	ND	2.77 ± 0.36 ^a^	ND	ND
Cis-Anethol	33.93	1721	31.02 ± 0.90 ^a^	79.40 ± 0.63 ^b^	20.92 ± 0.40 ^c^	74.61 ± 1.38 ^d^	ND	15.68 ± 1.86 ^e^
Total			32.01 ± 0.92 ^a^	82.21 ± 1.56 ^b^	20.92 ± 0.40 ^c^	77.38 ± 1.74 ^d^	ND	15.68 ± 1.86 ^e^
Terpenes								
α-Pinene	10.86	968	1.59 ± 0.04 ^b^	2.53 ± 0.26 ^b^	0.97 ± 0.01 ^b^	12.21 ± 0.75 ^a^	12.19 ± 0.62 ^a^	11.62 ± 0.70 ^a^
g-Terpinene	18.2	1121	1.62 ± 0.14 ^a^	5.88 ± 0.11 ^b^	0.72 ± 0.01 ^c^	3.54 ± 0.18 ^d^	ND	ND
Myrcene	15.39	1167	2.60 ± 0.16 ^a^	5.68 ± 0.23 ^b^	ND	5.05 ± 0.04 ^c^	ND	ND
(4R)-1-methyl-4-(prop-1-en-2-yl)cyclohex-1-ene	16.64	1114	9.07 ± 0.14 ^c^	20.89 ± 0.29 ^a^	5.43 ± 0.27 ^d^	18.25 ± 0.49 ^b^	4.28 ± 0.01 ^e^	5.84 ± 0.01 ^d^
3-Carene	14.95	1153	ND	6.7 ± 0.19 ^a^	ND	5.4 ± 0.01 ^b^	4.74 ± 0.24 ^c^	3.43 ± 0.18 ^d^
Total			14.88 ± 0.48 ^c^	41.68 ± 1.08 ^a^	7.12 ± 0.29 ^d^	44.45 ± 0.48 ^a^	21.21 ± 0.87 ^b^	20.89 ± 0.89 ^b^
Others								
2,6-Dimethylpyrazine	21.13	1275	0.36 ± 0.00 ^a^	ND	ND	ND	ND	ND
2,3,5,6-Tetramethylpyrazine	25.46	1395	0.25 ± 0.00 ^a^	0.39 ± 0.04 ^b^	ND	1.16 ± 0.11 ^c^	ND	ND
2-Acetyl pyrrole	28.72	1614	0.89 ± 0.05 ^a^	1.11 ± 0.00 ^a^	1.16 ± 0.00 ^a^	0.81 ± 0.00 ^a^	ND	ND
Naphthalene	33.06	1785	0.92 ± 0.10 ^a^	ND	ND	ND	ND	ND
Total			2.41 ± 0.15 ^a^	1.50 ± 0.04 ^b^	1.16 ± 0.00 ^c^	1.97 ± 0.11 ^d^	ND	ND

LRI: linear retention index. a–f Means within the same row with different superscript showing significant differences (*p* < 0.05). ND: volatile compounds not detected. CAS: CAS Registry Number. Control: non-high pressure and high-temperature and non-irradiated bacon; HTHP: high pressure and temperature treated bacon; 3 kGy: 3 kGy-dose irradiated bacon; 5 kGy: 5 kGy-dose irradiated bacon; 7 kGy: 7 kGy-dose irradiated bacon; 9 kGy: 9 kGy-dose irradiated bacon. R: Results were expressed as the mean values ± standard error (SE) (*n* = 3).

**Table 2:** Odour activity value (OAV) of volatile compounds of cooked bacon with different sterilizations.

Name	OT (μg L^−1^ of Water)	HTHP	Control	3 kGy	5 kGy	7 kGy	9 kGy
Isobutyl alcohol	0.500	0.764	2.882	2.222	3.610	8.576	19.136
Isoamyl alcohol	1.000	10.351	33.520	30.238	43.602	56.037	84.850
Cineole	5.000	4.339	8.907	8.120	8.720	4.666	16.269
Hexyl alcohol	1.600	1.086	1.598	1.501	2.092	0.952	1.048
1-Octen-3-ol	0.010	196.200	234.700	164.700	241.200	50.100	–
Linalool	0.005	4672.000	8253.000	3292.400	6544.400	625.200	1389.600
1-Octanol	0.190	5.121	–	3.489	7.574	–	–
Furfuryl alcohol	2.000	2.125	2.498	3.486	2.599	1.663	2.130
(S)-(-)-α-terpineol	0.28	10.611	19.511	5.425	12.254	–	–
Isovaleraldehyde	0.002	–	–	–	525.000	1390.000	10,639.583
Octanal	0.080	34.100	24.863	34.675	48.950	15.988	–
1-Nonanal	0.004	1754.773	1793.636	937.500	2231.136	470.455	485.909
Guaiacol	0.020	450.450	675.950	580.900	736.250	154.550	146.350
o-Cresol	0.650	6.355	8.303	6.109	9.486	1.389	–
Phenol	4.000	1.750	2.301	2.494	2.666	0.623	1.053
4-Ethyl-2-methoxyphenol	0.044	77.091	112.386	–	113.318	11.045	–
2-Pentanone	0.050	–	–	32.180	33.620	32.480	41.840
2-Heptanone	0.140	15.436	5.236	4.686	6.993	3.700	–
3-Hydroxy-2-butanone	8.000	0.660	–	1.327	1.626	5.625	4.315
Toluene	0.527	–	–	2.537	5.355	4.899	7.594
4-Allylanisole	0.035	28.314	80.400	–	79.171	–	–
2,3,5,6-Tetramethylpyrazine	1.000	0.254	0.387	–	1.155	–	–

OT: odour thresholds in water; OAV not calculation.

## Data Availability

Data is contained within the article.
